# Participation in the New Maudsley Model Workshops is Associated With Reductions in Caregiver Burden in Eating Disorders

**DOI:** 10.1002/erv.70111

**Published:** 2026-04-10

**Authors:** Cristiano Dani, Eleonora Rossi, Emanuele Cassioli, Giulia Selvi, Livio Tarchi, Francesco Rotella, Sandra Moretti, Maria Rita Troiani, Giulio D'Anna, Ilenia Giunti, Stefano Lucarelli, Janet Treasure, Valdo Ricca, Giovanni Castellini

**Affiliations:** ^1^ Department of Neurosciences, Psychology, Drug Research and Child Health University of Florence Florence Italy; ^2^ Careggi University Hospital Florence Italy; ^3^ Department of Health Sciences University of Florence Florence Italy; ^4^ UFC Eating Disorders Azienda USL Toscana Centro Florence Italy; ^5^ Centre for Research in Eating and Weight Disorders Institute of Psychiatry, Psychology and Neuroscience King's College London UK

**Keywords:** anorexia nervosa, caregiver burden, caregivers, New Maudsley Model, parents

## Abstract

**Objective:**

Caregivers of individuals with eating disorders (ED) frequently experience psychological distress, expressed emotion, and accommodation behaviours, contributing to caregiver burden. The New Maudsley Model (NMM) is a structured intervention designed to improve caregivers' emotional regulation, communication, and coping strategies. This study examined changes in caregiver burden associated with participation in NMM workshops and explored the temporal relationship between psychological distress and ED symptom impact.

**Method:**

A total of 447 caregivers participated, with 189 completing baseline and post‐intervention assessments, including psychological distress (DASS‐21), accommodation (AESED), expressed emotion (FQ), and ED symptom impact (EDSIS). Linear mixed models and structured equation modelling (SEM) were computed for longitudinal analyses.

**Results:**

Significant reductions were observed in anxiety, expressed emotion, accommodation, and ED symptom impact, following NMM workshops. Higher baseline psychological distress was associated with larger subsequent reductions in distress over time, while ED symptom impact was less modifiable over time, appearing influenced by family dynamics. Baseline ED symptom impact was associated with subsequent changes in caregiver distress.

**Discussion:**

These findings highlight the potential clinical utility of NMM workshops within standard ED care, emphasising the need to target caregiver beliefs, emotional responses, and coping behaviours to improve caregiver's well‐being and possibly support better ED outcomes.

## Highlights

- The New Maudsley Model (NMM) workshops were associated with reductions in caregiver burden, with improvements observed in anxiety, expressed emotion, accommodation behaviours, and key domains of ED symptom impact.
- Longitudinal modelling revealed a relationship between psychological distress and perceived ED symptom impact, supporting a feedback mechanism within caregiver burden.
- Findings highlight the clinical relevance of addressing caregiver disease representations and emotional responses as key targets for personalised, skills‐based interventions.

## 1 Introduction and Aims

Eating disorders (ED) are serious and complex psychiatric disorders, characterised by severe disturbances in eating behaviours and body image perception (Treasure et al. 2020). ED typically emerge during adolescence or early adulthood and are often marked by a chronic course, with many individuals experiencing prolonged disease duration and a high risk of relapse over time (Keski‐Rahkonen and Mustelin 2016; Solmi et al. 2024). Given the early onset and persistent nature of these disorders, family members often play a crucial role in caregiving (Wilksch 2023).

The role of caregiving for individuals with ED has often been associated with significant emotional strain, feelings of guilt, helplessness, and high levels of psychological distress, a phenomenon commonly described as “caregiver burden” (Hibbs et al. 2015). Building on these premises, caregiver burden in ED might be highly heterogeneous and influenced by multiple factors including psychological distress, caregiving skills, duration and intensity of care, and relationship (Pehlivan et al. 2024). Higher levels of burden have been particularly noted among caregivers with a personal story of ED, those assuming primary caregiving roles, and those supporting individuals with AN, who tend to exhibit greater psychological distress and accommodation behaviours, that may indeed contribute to the maintenance of the disease (Stefanini et al. 2019). Emerging evidence has indicated that providing care for individuals with ED may exert considerable psychological effects on caregivers, often resulting in elevated levels of stress, anxiety, depression, and feelings of helplessness (Zeiler et al. 2025). These challenges may culminate in post‐traumatic stress symptoms, particularly following the inpatient admission (Timko et al. 2023).

Notably, greater emotional sensitivity and fear have been also associated also higher accommodation in caregivers of individuals with ED (Kumar et al. 2023). Furthermore, elevated levels of expressed emotion within families, particularly emotional overinvolvement and criticism, have been linked to the persistence of ED symptoms and poorer treatment outcomes (Rhind et al. 2016; Zeiler et al. 2025). Conversely, supportive involvement from family members has been associated with more favourable recovery trajectories (Kurnik Mesarič et al. 2024; Wetzler et al. 2020).

From a theoretical perspective, these caregiver burden dimensions can be conceptualised as dynamically interrelated components of a broader caregiving stress‐response process, rather than as independent constructs. In particular, the perceived impact of ED symptoms may represent a persistent caregiving stressor, while psychological distress, expressed emotion, and accommodation behaviours reflect emotional and behavioural responses that may change over time (Di Lorenzo et al. 2025; Donkin et al. 2025). Importantly, these processes may develop both within individuals (e.g., how caregivers cognitively and emotionally respond to illness‐related stressors) and between individuals, as caregiver distress and coping responses can themselves become potential stressors for the patient, and vice versa. Because existing literature does not establish a clear causal ordering between these dimensions, the present study adopts a longitudinal approach that allows for the examination of temporal coupling without imposing a priori directionality.

Nevertheless, recent evidence has suggested that caregiver burden often remains significant despite the implementation of therapeutic interventions, with lasting effects on family wellbeing irrespective of the treatment modality applied (Pedersen et al. 2024). This underscored the importance of specifically addressing caregiver needs as a means to support both individual recovery and overall family functioning (Treasure et al. 2021).

In this context, skills‐based parental interventions have demonstrated effectiveness in reducing caregiver burden, psychological distress, and emotional overinvolvement among those caring for adolescents with AN (Hibbs et al. 2015; Zeiler et al. 2023).

Models such as the New Maudsley Model (NMM) have been specifically developed to provide caregivers practical tools to regulate emotional responses, enhance communication, and support their family members recovery process more effectively (Treasure et al. 2015). A qualitative study showed that individuals with AN perceived the NMM workshops as beneficial in improving communication, reducing emotional reactivity, and strengthening supportive relationships with carers, with positive effects on relapse prevention and daily functioning (Goddard et al. 2011). The integration of caregiver‐focused interventions into treatment pathways may represent a crucial step toward more personalised and systemically informed care models for ED (Treasure et al. 2021). Recent evidence shows that adding NMM workshops to CBT‐E for adults with AN is associated with greater clinical improvements, underscoring the value of structured caregiver involvement (Dani et al. 2025). Although several studies and meta‐analyses have evaluated caregiver interventions derived from the New Maudsley or cognitive‐interpersonal framework and reported improvements in caregiver distress, burden, and expressed emotion (Hibbs et al. 2015; Quiles Marcos et al. 2026), less is known about how different dimensions of caregiver burden interact dynamically over time. In particular, the temporal relationship between caregivers' psychological distress and the perceived impact of ED symptoms has not yet been examined within a longitudinal dynamic modelling framework.

In this context, the present study aimed to examine changes in caregiver burden and its key dimensions among caregivers of individuals with ED in association with participation in NMM training workshops. Specifically, changes in psychological distress, accommodation behaviours, expressed emotion, and perceived ED symptom impact were assessed before and after the intervention. In addition, the study used structural equation modelling (SEM) to explore temporal associations between baseline levels and subsequent changes in psychological distress and perceived ED symptom impact, focusing on patterns of temporal coupling among caregiver burden domains.

## 2 Method

### 2.1 Study Design

The study enroled caregivers of individuals diagnosed with ED admitted to the Eating Disorders Unit of Azienda USL Toscana Centro, Florence, Italy between 2020 and 2024. Caregivers were consecutively identified among family members of individuals with ED admitted to the unit and were invited to participate at the beginning of the New Maudsley Model (NMM) training workshops.

The study protocol was approved by the Institutional Ethics Committee (Comitato Etico Regione Toscana ‐ Area Vasta Centro (CEAVC); reference number: OSS.14.162). All participants received a complete explanation of the study aims and procedures and provided written informed consent prior to participation, in accordance with the Declaration of Helsinki.

The inclusion criteria were as follows: participation to NMM training workshops; age over 18 years; being a caregiver of an individual diagnosed with ED at the time of recruitment, according to the Diagnostic and Statistical Manual of Mental Disorders, Fifth Edition (DSM‐5; American Psychiatric Association 2013); the provision of written informed consent. The exclusion criteria included: illiteracy, intellectual disability, or any other condition that could impair the proper understanding of the study protocol and completion of the questionnaires.

Of the 477 caregivers initially enroled, 23 participants (4.8%) dropped out during the intervention. Of the remaining caregivers, 447 were assessed at baseline (T0), and 189 completed the post‐intervention assessment (T1), while the others were lost to follow‐up.

Participant flow from recruitment to final analyses is shown in Supporting Information S1: Figure S1.

### 2.2 Study Population

All the participants attended the NNM training workshops for caregivers of individuals with ED. The sample comprised 447 caregivers, including 255 mothers (57.0%) and 192 fathers (43.0%). The mean age of the caregivers was 51.87 years (SD = 5.78). To assess the impact of the intervention on caregiver burden and family functioning, the same set of psychometric questionnaires was administered to all participants at both baseline (T0) and post‐intervention (T1).

Caregivers were supporting 447 female individuals with ED, each meeting DSM‐5 diagnostic criteria at the time of recruitment. Specifically, 344 individuals (77.0%) were diagnosed with anorexia nervosa (AN) and 103 (23.0%) with bulimia nervosa (BN). At baseline (T0), the mean illness duration of the affected individuals was 3.39 years (SD = 1.73), and the mean age was 16.99 years (SD = 2.51).

The 447 caregivers referred to 280 unique individuals with ED, corresponding to 280 family units; some family units were represented by a single caregiver, whereas others were represented by two caregivers (mother and father).

### 2.3 Data Collection

Each participant participated in a diagnostic interview to confirm their eligibility for inclusion in the study. Data collection was conducted by clinicians with expertise in ED who were not involved in the delivery of the NMM training workshops. Data were collected at the beginning (T0) and at the end (T1) of the NMM training workshops (seven sessions; ∼8–10 weeks after baseline).

The following questionnaires were administered to assess caregiver burden and family functioning:

- The Depression, Anxiety, and Stress Scale (DASS‐21; Bottesi et al. 2015; Henry and Crawford 2005) is a 21‐items questionnaire scored on a four‐point Likert scale. It assesses three psychological domains—depression (D), anxiety (A) and stress (S)—through three subscales. In the recruited population, the questionnaire demonstrated good internal consistency: D (*α* = 0.82), A (*α* = 0.78), S (*α* = 0.84).
- The Accommodation and Enabling Scale for Eating Disorders (AESED; Fasolato et al. 2025; Sepulveda et al. 2009) is a 33‐items questionnaire using a five‐point Likert scale to assess the extent to which caregivers tolerate or facilitate ED‐related behaviours at home. Specifically, it consists of the following subscales: avoidance and routine modification (AMR), reassurance‐seeking (RS), meal‐related rituals (MR), family control (FC), “turning a blind eye” (TBE), and Overall. Cronbach's alpha for the subscales were the following: AMR (*α* = 0.83); RS (*α* = 0.86); MR (*α* = 0.80); FC (*α* = 0.84), TBE (*α* = 0.74), overall (*α* = 0.89).
- The Eating Disorder Symptom Impact Scale (EDSIS; Sepulveda et al. 2008) is a 20‐item questionnaire using a five‐point Likert scale to assess the impact of eating disorder symptoms on caregivers. It includes the following subscales: nutrition‐related distress (N), guilt (G), dysregulated behaviours (DB), and social isolation (SI). The internal consistency for the subscales was (Cronbach's *α*): N 0.78, G 0.82, DB 0.76, SI 0.83, Total 0.88.
- The Family Questionnaire (FQ; Ponti et al. 2018; Wiedemann et al. 2002) is a self‐report measure designed to assess expressed emotion (EE) in caregivers of individuals with eating disorders. Expressed emotion refers to the degree of emotional involvement and critical attitudes a caregiver exhibits toward the individual with the disorder. The FQ consists of 20 items scored on a four‐point Likert scale, assessing these specific domains of EE: critical comments (CC), emotional over‐involvement (EOI), and Total Score. Results indicated good internal reliability across all subscales: CC (*α* = 0.86), EOI (*α* = 0.78), Total (*α* = 0.87).

### 2.4 Intervention

All the participants of the study participated in NMM training workshops, which aimed to equip them with practical tools and strategies to better support their family members with an ED (Treasure et al. 2015). This programme consisted of seven structured sessions, each lasting two hours, delivered weekly over approximately 8–10 weeks, and was conducted by team members with expertise in the fields of ED and specifically trained for NMM. Workshops were delivered in small groups, with an average of approximately 15–20 caregivers per group. The training follows a theoretical model of carer coping and caregiving behaviour, incorporating practical strategies to reduce stress and enhance adaptive responses such as emotion regulation, communication skills, and boundaries definition. Central to the model is the adoption of a “C” caregiving style, which promotes compassion, consistency, and collaboration (Treasure et al. 2015). Carers are encouraged to support their family members while simultaneously promoting autonomy and reducing overinvolvement (Treasure et al. 2015).

The training workshops facilitators suggested that participants purchase the written NMM manual in Italian (Stefanini et al. 2024).

A more detailed description of the NMM training workshops is provided in the Supplementary Materials.

### 2.5 Statistical Analyses

Continuous variables were reported as mean and standard deviation. The longitudinal assessment of changes in caregiver burden and family functioning over time, as influenced by NMM training workshops participation, was conducted using linear mixed models with random intercepts. These models account for individual differences and allow for the examination of within‐subject changes while adjusting for baseline variability. Age was included as an adjustment variable to adjust for its potential confounding effects.

An a priori power analysis was conducted based on repeated‐measures ANOVA assumptions (two time points, correlation among repeated measures = 0.40, nonsphericity correction *ε* = 1). Assuming a small‐to‐moderate effect size (*f* = 0.20), an alpha level of 0.05, and a desired power of 0.80, the analysis indicated that a total sample size of 61 caregivers would be required to reliably detect longitudinal changes in caregiver burden‐related outcomes.

Additionally, to account for the hierarchical structure of the data, linear mixed‐effects models included caregiver and individual with ED's identifiers as grouping factors, reflecting the nested structure of the dataset, with repeated measurements nested within caregivers and caregivers nested within individuals with ED. This approach accounts for both within‐caregiver correlations across time and potential non‐independence among caregivers referring to the same individual with ED. Time was modelled as a slope to estimate changes across the two assessment points.

The present study also explored the dynamic relationship between family functioning over time. Longitudinal change between T0 and T1 was operationalised using observed difference scores. For each indicator, change scores (Δ) were computed as the difference between T1 and T0 values (T1–T0) and subsequently rescaled by dividing by 10 to improve numerical stability during model estimation. Change scores were included as observed variables representing longitudinal change between T0 and T1. This approach allows the modelling of longitudinal change and cross‐domain coupling effects between constructs within a SEM framework. By incorporating this framework within SEM, individual trajectories of distress (DASS‐21) and ED symptoms impact (EDSIS) were assessed, while simultaneously testing how changes in one construct influence shifts in the other over time. Unlike traditional regression‐based longitudinal models, the SEM model enables a detailed examination of multiple subdomains within each construct like distress (DASS‐21) and ED symptoms impact (EDSIS), allowing for the simultaneous modelling of both domain‐specific and global change processes. Critically, the model incorporates cross‐domain coupling effects, facilitating the evaluation of whether baseline levels in one domain (e.g., distress) predict subsequent changes in the other (e.g., ED symptoms), and vice versa. This analytic framework offers a comprehensive representation of the bidirectional and multidimensional nature of psychological change over time.

Within the SEM model, psychological distress (DASS‐21) and perceived ED symptom impact (EDSIS) were treated as core dynamic constructs rather than as fixed predictors or outcomes. Baseline latent variables were specified as predictors of their own subsequent change (autoregressive effects) and of change in the other domain (cross‐domain coupling effects), while change scores (Δ) were specified as outcomes. Baseline constructs were defined using a multiple‐indicator approach, with DASS‐21 modelled through its subscales (depression, anxiety, and stress) and EDSIS through its domains (nutrition‐related distress, guilt, dysregulated behaviours, and social isolation), whereas latent change factors were indicated by observed difference scores (Δ) computed for each indicator between T1 and T0. In addition, changes in family expressed emotion (FQ) and accommodation behaviours (AESED) were included in the model as observed change variables to account for concurrent changes in the family environment and caregiving responses. These variables were modelled as auxiliary predictors of change within the system rather than as core latent constructs in the coupling model. This analytic specification reflects the conceptual framework outlined in the Introduction, which views caregiver burden dimensions as dynamically interrelated processes rather than as unidirectional causal pathways.

The final model was estimated using Full Information Maximum Likelihood (FIML) and robust (Huber‐White) standard errors. Model fit was determined based on standard indices: *χ* ^2^ (should be non‐significant), Comparative Fit Index (CFI ≥ 0.95), Tucker–Lewis Index (TLI ≥ 0.95), Root Mean Square Error of Approximation (RMSEA ≤ 0.06), and Standardized Root Mean Square Residual (SRMR ≤ 0.08) (Kenny and McCoach 2003; Schreiber et al. 2006).

Sociodemographic and illness‐related variables were examined in sensitivity analyses. In the supplementary mixed‐effects models, the age of the individual with ED, illness duration, ED diagnostic category (AN vs. BN), and the caregiver sex × Time interaction were tested as additional predictors of longitudinal change. In addition, within the SEM framework, the age of the individual with ED, illness duration, ED diagnostic category (AN vs. BN), and caregiver sex were examined as time‐invariant covariates, entered as predictors of baseline latent factors and change scores. As the inclusion of these variables did not materially alter the pattern or magnitude of the main coupling effects, they were not included in the final model specification to preserve model parsimony and maintain focus on the core dynamic mechanisms of change.

The statistical analyses were performed with R statistical software v4.5.1, with the following libraries: dplyr (Wickham et al. 2023), nlme (Pinheiro et al. 2021), lavaan (Rosseel 2012), semPlot (Epskamp 2015).

## 3 Results

### 3.1 Longitudinal Effect of NMM Groups on Caregiver Burden

The analyses of longitudinal changes from baseline to end‐of‐workshop assessments revealed significant improvements across multiple measures. Results are shown in Table 1.

**TABLE 1:** Longitudinal effect of NMM groups on caregiver burden from baseline (T0) to end‐of‐workshops (T1).

	T0 (*n* = 447)	T1 (*n* = 189)	Beta	*p*‐value
DASS‐21 D	11.03 ± 8.10	9.53 ± 6.89	−1.19	0.095
DASS‐21 A	6.91 ± 6.76	4.98 ± 5.60	−2.00	**<** **0.001**
DASS‐21 S	14.63 ± 8.30	14.06 ± 8.13	−0.80	0.339
FQ CC	1.89 ± 0.49	1.85 ± 0.59	−0.09	**0.026**
FQ EOI	2.52 ± 0.50	2.45 ± 0.47	−0.08	0.053
FQ TOT	2.25 ± 0.47	2.17 ± 0.41	−0.08	**0.022**
AESED AMR	20.67 ± 9.68	18.70 ± 8.78	−2.45	**<** **0.001**
AESED RS	10.69 ± 7.35	9.20 ± 6.50	−2.32	**<** **0.001**
AESED MR	20.67 ± 9.68	18.70 ± 8.78	−0.55	0.252
AESED CF	9.11 ± 4.22	7.70 ± 4.25	−1.71	**<** **0.001**
AESED TBE	2.62 ± 3.26	2.16 ± 2.69	−0.81	**0.002**
AESED overall	46.42 ± 20.06	40.90 ± 18.15	−7.65	**<** **0.001**
EDSIS N	16.01 ± 20.06	13.50 ± 18.16	−2.87	**<** **0.001**
EDSIS DB	6.21 ± 4.63	5.85 ± 4.19	−0.84	**0.010**
EDSIS G	9.03 ± 4.56	7.72 ± 3.99	−1.21	**0.002**
EDSIS SI	4.54 ± 3.82	4.44 ± 3.84	−0.49	0.095
EDSIS total	34.84 ± 15.12	31.12 ± 14.30	−5.25	**<** **0.001**

*Note:* Caregiver age‐adjusted linear mixed models are presented. Beta coefficients are reported along their *p*‐values. Significant values (*p* < 0.05) are shown in bold.

Abbreviations: A, Anxiety; AESED, Accommodation and Enabling Scale for Eating Disorders; AMR, Avoidance and Modifying Routine; CC, Critical Comments; CF, Control of Family; D, Depression; DASS‐21, Depression, Anxiety and Stress Scale‐21; DB, Dysregulated Behaviour; EDSIS, Eating Disorders Symptom Impact Scale; EOI, Emotional Overinvolvement; FQ, Family Questionnaire; N, Nutrition; RS, Reassurance Seeking; MR, Meal Ritual; S, Stress; SI, Social Isolation; TBE, Turning a Blind Eye; TOT, Total Score.

Regarding psychological distress (DASS‐21), a significant reduction over time was observed in anxiety, while no significant changes were found in depression and stress. Additionally, a significant decrease was observed in expressed emotion, as indicated by the total score of the FQ. Moreover, a significant reduction was found in the domain of critical comments, whereas emotional overinvolvement showed only a trend toward significance. In terms of accommodation (AESED), significant reductions were observed across most subscales, including avoidance and modifying routine, reassurance seeking, control of family, turning a blind eye, and the overall score. Similarly, ED symptom impact (EDSIS) showed significant reductions in the domains of nutrition‐related distress, dysregulated behaviour, guilt, and total score.

### 3.2 Multivariate Dynamic Associations Across Caregiver Burden Domains

**FIGURE 1:**
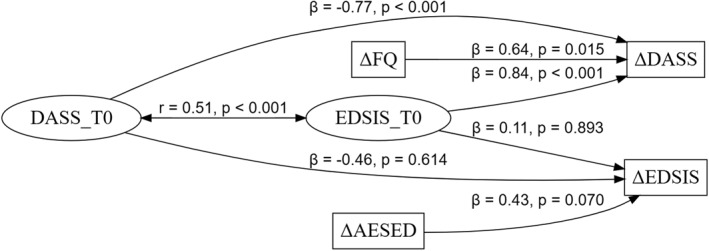
Conceptual path diagram of SEM model examining dynamic associations among caregiver burden domains. Baseline latent constructs include psychological distress (DASS_T0) and perceived ED symptom impact (EDSIS_T0). Change scores (Δ variables) represent pre‐to‐post intervention differences in psychological distress (ΔDASS), ED symptom impact (ΔEDSIS), expressed emotion (ΔFQ), and accommodation behaviours (ΔAESED). Single‐headed arrows indicate standardized regression paths (*β*) and associated *p*‐values between baseline variables and subsequent changes, as well as associations among change processes; double‐headed arrows indicate correlations (r) between baseline constructs. All coefficients are standardized estimates derived from the structural equation model. AESED, Accommodation and Enabling Scale for Eating Disorders; DASS‐21, Depression Anxiety and Stress Scale; EDSIS, Eating Disorder Symptom Impact Scale; FQ, Family Questionnaire.

The results indicated excellent model fit *χ* ^2^(75) = 57.324, *p* = 0.896, CFI = 1.000, TLI = 1.014, RMSEA = 0.000, and SRMR = 0.044. The conceptual structure of the SEM model is illustrated in Figure 1.

The structural relationships within the model revealed a significant autoregressive effect for psychological distress (DASS‐21), indicating that higher baseline distress (DASS_T0) was associated with a steeper subsequent decline in psychological distress over time (ΔDASS‐21; *β* = −0.77, *p* < 0.001). In contrast, the autoregressive effect of ED symptom impact (EDSIS_T0) on changes in ED symptom impact over time (ΔEDSIS) was not significant (*β* = 0.11, *p* = 0.893). Moreover, baseline ED symptom impact (EDSIS_T0) significantly predicted changes in psychological distress over time (ΔDASS‐21), indicating that a higher initial burden of ED symptoms is associated with increased psychological distress over time (*β* = 0.84, *p* < 0.001). Conversely, baseline psychological distress (DASS_T0) did not significantly predict changes in ED symptom impact over time (ΔEDSIS).

Furthermore, family expressed emotion (FQ) was examined as a potential factor influencing symptom trajectories. Changes in overall family expressed emotion (ΔFQ) were significantly associated with changes in psychological distress (ΔDASS‐21; *β* = 0.64, *p* = 0.015), suggesting that shifts in family emotional climate, including criticism, emotional overinvolvement, or support, may contribute to fluctuations in psychological distress. However, the direct effect of changes in family expressed emotion (ΔFQ) on changes in the perceived impact of eating disorder symptoms (ΔEDSIS) was not significant. Similarly, changes in accommodation behaviours (ΔAESED) were not significantly associated with changes in perceived ED symptom impact (ΔEDSIS).

Significant covariances were observed among DASS subdomains (changes in stress, anxiety, and depressive symptoms) and ED symptoms (EDSIS) impact subdomains (changes in nutrition‐related distress, guilt, dysregulated behaviours, and social isolation), reinforcing the strong interconnections within each domain. In addition, baseline psychological distress (DASS_T0) and ED symptom impact (EDSIS_T0) were significantly correlated (*r* = 0.51, *p* < 0.001). Several cross‐domain relationships also emerged, including a significant association between higher depressive symptoms at baseline and increased guilt‐related impact, suggesting that greater depression severity is linked to a higher global burden of ED symptoms. Social isolation related to ED symptoms at baseline was significantly correlated with depressive symptoms, indicating that social withdrawal is both a consequence and a maintaining factor of psychological distress. Additionally, baseline stress levels were negatively associated with nutrition‐related impact, suggesting that individuals experiencing higher stress may exhibit fewer overt nutritional impairments, possibly due to differences in coping mechanisms or compensatory behaviours. A complete representation of the model is provided in Supporting Information S1: Figure S2.

### 3.3 Sensitivity Analyses

Sensitivity analyses were conducted to further characterise the sample and assess the robustness of the findings. Baseline comparisons between mothers and fathers indicated higher levels of psychological distress and caregiver burden among mothers across several domains, including depression, anxiety, stress, expressed emotion, and perceived ED symptom impact (Supporting Information S1: Table S1). The analyses examining the potential influence of additional sociodemographic and illness‐related variables, including the age of the individual with ED, illness duration, ED diagnostic category (AN vs. BN), and the caregiver's sex × Time interaction, did not reveal significant associations with longitudinal changes in caregiver outcomes (Supporting Information S1: Table S2).

In addition, these variables were examined as time‐invariant covariates within the SEM framework. The adjusted model showed good fit to the data: *χ* ^2^(117) = 135.57, *p* = 0.115; CFI = 0.983; TLI = 0.973; RMSEA = 0.033, SRMR = 0.065. The inclusion of these covariates did not materially alter the pattern or magnitude of the main coupling effects observed in the primary model.

Finally, baseline comparisons between caregivers who completed the post‐intervention assessment and those lost to follow‐up did not show significant differences across the main outcome measures, suggesting limited evidence of systematic attrition bias (Supporting Information S1: Table S3).

## 4 Discussion

The present study investigated the longitudinal associations of a caregiver‐focused intervention, the NMM training workshops, on psychological distress, accommodation behaviours, and the perceived impact of ED symptoms among caregivers of individuals with ED.

The findings indicated that caregivers of individuals with ED reported psychological distress, expressed emotion, accommodation behaviours, and perceived impact of ED symptoms at baseline, consistent with patterns described in previous research (Sepúlveda et al. 2012; Stefanini et al. 2019; Zabala et al. 2009). Moreover, participation in NMM training workshops was associated with significant improvements across multiple domains of caregiver burden. In particular, reductions in anxiety, expressed emotions, dimensions of accommodation and the impact of ED symptoms, were consistent with the potential of structured, skills‐based programs to alleviate family strain and enhance caregiver psychological well‐being (Bijsterbosch et al. 2025; Treasure et al. 2021). This result is clinically relevant, as the observed declines in accommodating behaviours, such as routine modification and reassurance‐seeking, may emphasise the potential role of behavioural accommodation in maintaining ED symptoms (Pedersen et al. 2024).

Beyond group‐level effects, the results offered novel insights into the dynamic nature of psychological burden within the caregiving context. Specifically, results indicated significant unidirectional influences between caregivers' psychological distress and the perceived impact of ED symptoms: baseline levels of perceived ED symptom impact predicted subsequent changes in caregivers' psychological distress over time, whereas baseline distress did not significantly predict changes in perceived ED symptom impact. Although no bidirectional effect emerged, the observed association may still reflect a vulnerability pathway, whereby sustained exposure to symptom‐related demands contributes to heightened emotional burden (Marchetti and Sawrikar 2024). These findings are in line with recent network analyses showing the interconnection between emotional overinvolvement and accommodation in caregivers and disease symptomatology, playing a central role in maintaining disease‐related distress (Monteleone et al. 2023, 2024).

Additional sensitivity analyses provided further context for these findings. Baseline comparisons suggested higher levels of psychological distress and caregiver burden among mothers compared with fathers, in line with previous literature (Zeiler et al. 2023). However, age of the individual with ED, illness duration, diagnostic category, and the caregiver sex × Time interaction were not associated with longitudinal changes in caregiver outcomes. Furthermore, no significant baseline differences were observed between participants who completed the post‐intervention assessment and those lost to follow‐up.

The present results raise the possibility that, given the high caregiver burden and its reduction following the intervention, approaches such as NMM may also be relevant for ED outcomes (Dani et al. 2025). This is consistent with family‐based models of ED maintenance, which highlight the importance of the caregiver emotional climate and expressed emotion in influencing the course of the disorder (Hibbs et al. 2015; Treasure et al. 2015).

Interestingly, the autoregressive effect for psychopathological distress was significant, indicating that higher initial symptom severity was associated with greater reductions over time, possibly reflecting a more pronounced effect of the intervention among those with higher baseline distress. In contrast, the absence of a significant autoregressive effect for ED symptom impact suggests that perceived symptom impact may be less sensitive to change and more influenced by factors such as family dynamics or ED treatment effects, particularly those aimed at improving caregiver emotion regulation and disease understanding (Pehlivan et al. 2024).

Disease representation by caregivers might be among the most resistant elements to shift without targeted intervention, yet remaining key therapeutic targets for improving caregiver well‐being and engagement in supportive behaviours (Marchetti and Sawrikar 2024).

Notably, changes in expressed emotion were significantly associated with shifts in psychological distress, but not directly with changes in ED symptom impact. This pattern suggests that the family emotional environment may exert its influence primarily through caregivers' emotional states, rather than directly modifying symptom appraisals (Treasure and Livanou 2024). Moreover, these findings support existing literature emphasising the role of expressed emotion in shaping caregiver burden and psychological outcomes (Matthews et al. 2018). Caregivers often perceive ED as uncontrollable, incomprehensible, and chronic, leading to emotions such as fear, guilt, and helplessness; these representations may contribute to caregiver distress and impair coping, highlighting the importance of targeting caregivers' disease beliefs within interventions (Marchetti and Sawrikar 2024). The prominent role of guilt and social isolation observed in the model may be interpreted within the broader context of ED‐related stigma. Experiences of stigma across social, healthcare, and family settings have been shown to be associated with social withdrawal and ambivalence toward help‐seeking (O’Connor et al. 2022), while broader structural under‐recognition of ED within mental health research and care systems may further contribute to delayed support (Schmidt et al. 2025). By addressing both emotional distress and maladaptive responses such as accommodation and emotional reactivity, these interventions may disrupt reinforcing cycles that maintain caregiver burden and, in turn, support more effective recovery within a healthier family environment (Herpertz‐Dahlmann et al. 2021; Kurnik Mesarič et al. 2024). This underscores the value of integrating caregiver‐focused programs like the NMM training workshops into standard treatment protocols for ED (Dani et al. 2025; Davey et al. 2023).

Nevertheless, several limitations must be considered. First, the study employed a longitudinal design without a randomized control group, limiting the strength of causal inferences. The absence of a control group reflects the naturalistic implementation of the NMM workshops within routine clinical care, where the intervention was offered as part of standard caregiver support. Although significant changes were observed following participation in the NMM workshops, these cannot be uniquely attributed to the intervention and may reflect alternative explanations, including natural adaptation to caregiving demands over time, or the influence of concurrent treatments. Second, a significant limitation is the reduced number of caregivers completing the follow‐up assessment (T1) compared to the initial sample. This reduction was due both to participant dropout during the intervention and to loss to follow‐up, which may introduce attrition‐related selection bias and limit the generalisability of the findings, as caregivers who completed the intervention and follow‐up assessments may differ systematically from those who discontinued participation or were lost to follow‐up. However, baseline comparisons between caregivers who completed the post‐intervention assessment and those lost to follow‐up did not show significant differences across the main outcome measures (Supporting Information S1: Table S3), suggesting limited evidence of systematic attrition bias. In addition, the availability of detailed sociodemographic information on caregivers was limited, which may constrain the interpretation of potential sources of variability in caregiving experiences. To strengthen the evidence base, future research should replicate these findings using randomized controlled designs and extended follow‐up periods to evaluate the durability of intervention‐related changes. In addition, although the EDSIS showed good internal consistency in the present sample, it has not yet been formally validated in Italian, and this should be considered when interpreting findings involving perceived ED symptom impact.

In conclusion, the present study provides evidence consistent with the potential effectiveness of NMM training workshops in reducing caregiver burden in the context of ED. Beyond confirming the intervention associated changes, the findings underlined the dynamic relationship between caregiver distress and perceived symptom impact, emphasizing the need to address the emotional interdependence that shapes caregiving experiences (Treasure and Livanou 2024). These results point to the importance of tailoring intervention strategies not only to symptom severity but also to caregivers' individual emotional profiles, illness representations, and coping patterns. Improving routine clinical care with such personalized, skills‐based approaches may represent a critical advancement in supporting families and may contribute to more sustainable recovery trajectories for individuals with ED (Schmidt et al. 2025).

## Author Contributions

**Cristiano Dani:** conceptualization, data curation, methodology, formal analysis, writing – original draft, writing – review and editing. **Eleonora Rossi:** conceptualization, supervision, writing – review and editing. **Emanuele Cassioli:** conceptualization, methodology, supervision, writing – review and editing. **Giulia Selvi:** data curation, writing – review and editing. **Livio Tarchi:** supervision, writing – review and editing. **Francesco Rotella:** validation, writing – review and editing. **Sandra Moretti:** supervision, writing – review and editing. **Maria Rita Troiani:** data curation, validation, writing – review and editing. **Giulio D'Anna:** supervision, writing – review and editing. **Ilenia Giunti:** validation, writing – review and editing. **Stefano Lucarelli:** validation, writing – review and editing. **Janet Treasure:** validation, visualization, writing – review and editing. **Valdo Ricca:** validation, visualization, writing – review and editing. **Giovanni Castellini:** conceptualization, supervision, validation, visualization, writing – review and editing.

## Funding

This work was supported by #NEXTGENERATIONEU (NGEU) and funded by the Ministry of University and Research (MUR), National Recovery and Resilience Plan (NRRP), project MNESYS (PE0000006)–A Multiscale integrated approach to the study of the nervous system in health and disease (DR. 1553 11.10.2022).

## Ethics Statement

The study protocol was approved by the Institutional Ethics Committee.

## Consent

Written informed consent was obtained from all participants prior to inclusion in the study.

## Conflicts of Interest

The authors declare no conflicts of interest.

## Supporting information


Supporting Information S1

**Figure S2:** Path diagram illustrating the structural equation model (SEM) examining dynamic associations across caregiver burden domains.

## Data Availability

The data that support the findings of this study are available from the corresponding author upon reasonable request.
