# Supplementary material for: Participation in the New Maudsley Model Workshops is Associated With Reductions in Caregiver Burden in Eating Disorders
Source: Eur Eat Disord Rev. 2026 Apr 10;34(5):1221–30. doi: 10.1002/erv.70111 (PMC13432577; doi:10.1002/erv.70111)
Supplement: Supplementary file 1 — Supporting Information S1 [file ERV-34-1221-s002.docx]

**Supplementary Material**

**New Maudsley Model (NMM) training workshops: overview**

The New Maudsley Model (NMM) training workshops are a structured, skills-based intervention for caregivers of individuals with eating disorders (ED; Stefanini et al., 2024; Treasure et al., 2015). The approach is grounded in a collaborative care framework and conceptualizes families as a key resource in treatment rather than as a causal factor in the disorder. The training workshops are delivered as an adjunct to clinical treatment and are not intended as a stand-alone therapeutic intervention.

The primary aims of the training workshops are to reduce caregiver anxiety and emotional distress, moderate high expressed emotion, limit accommodating or enabling behaviors, and strengthen caregivers’ emotional regulation, communication, and coping skills. The workshops focus on helping caregivers understand the psychological, interpersonal, and behavioral impact of eating disorders and to recognize automatic caregiving responses that may inadvertently maintain symptoms.

Format and delivery

The program consists of seven training workshops, including an introductory orientation session followed by six structured workshops. Training workshops are delivered by two professionals with expertise in ED and specific training in the New Maudsley Model. The number of participants is kept limited to facilitate interaction, reflection, and skills practice. Each workshop lasts approximately 90-120 minutes. The training workshops integrate psychoeducation, guided reflection, practical exercises, and role-playing. Emphasis is placed on applying skills to everyday caregiving situations and on promoting a supportive and collaborative learning environment.

Core content and skills

Across the training workshops, caregivers are introduced to a range of practical skills and techniques commonly used within the New Maudsley Model, including:

- Emotional regulation and self-care, aimed at managing fear, frustration, guilt, and helplessness, and reducing caregiver stress.

- Communication skills designed to reduce critical, hostile, or overinvolved responses and promote a compassionate, consistent, and collaborative caregiving stance (“C-style”). Communication strategies are informed by motivational interviewing, including reflective listening, open-ended questions, and collaborative goal setting.

- Managing ambivalence and resistance to change, using structured tools such as decisional balance exercises, rulers, and functional (ABC) models.

- Reducing accommodation and enabling behaviors, through increased awareness of habitual caregiving responses and gradual modification of behaviors that may reinforce eating disorder symptoms.

- Experiential learning and role-play, allowing caregivers to practice alternative responses to challenging situations and reflect on their effects.

Throughout the training workshops, caregivers are encouraged to reflect on their own emotional reactions, develop greater flexibility in caregiving behaviors, and support autonomy while maintaining appropriate boundaries. The training workshops aim to equip caregivers with a practical and adaptable skill set to support their loved one while preserving caregiver well-being.

Bibliography

Stefanini, M. C., Troiani, M. R., & Treasure, J. (2024). *Applicare il nuovo metodo Maudsley. Guida italiana alla conduzione di gruppi di familiari di persone con disturbo dell’alimentazione*. Hogrefe Editore Srl.

Treasure, J., Rhind, C., Macdonald, P., & Todd, G. (2015). Collaborative Care: The New Maudsley Model. *Eating Disorders*, *23*(4), 366–376. https://doi.org/10.1080/10640266.2015.1044351

**Figure S1. Flow diagram of recruitment process**

**
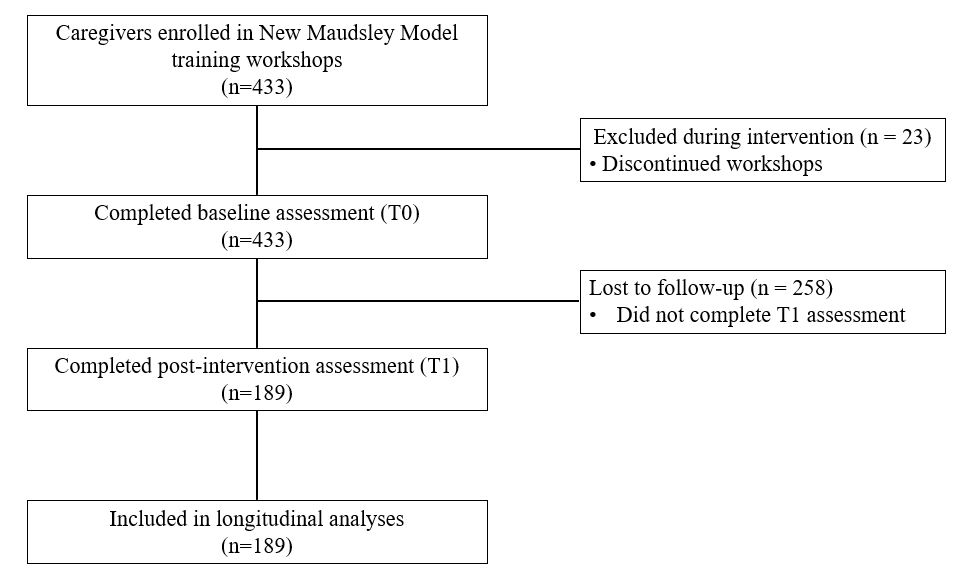
**

**Figure S2. Path diagram illustrating the structural equation model (SEM) examining dynamic associations across caregiver burden domains.**

*Note:* The diagram presents the complete structural equation model including baseline latent constructs, latent change factors, observed indicators, and estimated covariances. Baseline psychological distress (DASS_T0) is modeled as a latent factor indicated by the DASS-21 subscales (depression, anxiety, and stress), while baseline perceived ED symptom impact (EDSIS_T0) is indicated by the EDSIS domains (nutrition-related distress, guilt, dysregulated behaviours, and social isolation). Latent change factors represent pre-post changes in psychological distress (deltaDASS) and ED symptom impact (deltaEDSIS). Additional observed change variables include expressed emotion (deltaFQ) and accommodation behaviours (deltaAESED). Arrows represent structural paths and covariances estimated in the model. For clarity, standardized coefficients are not displayed.

Abbreviations: DASS-21: Depression Anxiety and Stress Scale; DASS21_D_T0: Depression subscale at baseline (T0); DASS21_A_T0: Anxiety subscale at baseline (T0); DASS21_S_T0: Stress subscale at baseline (T0); DASS_T0: latent factor for baseline psychological distress; delta_dass21: change in total DASS-21 score; delta_dass21_d: change in Depression score; delta_dass21_a: change in Anxiety score; delta_dass21_s: change in Stress score; EDSIS: Eating Disorder Symptom Impact Scale; EDSIS_N_T0: Nutrition subscale at baseline (T0); EDSIS_G_T0: Guilt subscale at baseline (T0); EDSIS_DB_T0: Dysregulated Behaviour subscale at baseline (T0); EDSIS_SI_T0: Social Isolation subscale at baseline (T0); EDSIS_T0: latent factor for baseline ED symptom impact; delta_edsis: change in total EDSIS score; delta_edsis_n: change in Nutrition impact; delta_edsis_g: change in Guilt impact; delta_edsis_db: change in Dysregulated Behaviour impact; delta_edsis_si: change in Social Isolation impact; delta_aesed_tot: change in AESED (Accommodation and Enabling Scale for Eating Disorders) total score; delta_fq_tot: Change in Family Questionnaire total score.

**Table S1. Baseline group differences between mothers and fathers in psychological distress and caregiver burden**

|  | Mothers  (n=255) | Fathers  (n=192) | F | p-value |
| --- | --- | --- | --- | --- |
| DASS-21 D | 12.39 ± 8.20 | 9.21 ± 7.62 | 17.51 | **<0.001** |
| DASS-21 A | 9.46 ± 7.10 | 4.86 ± 5.68 | 33.28 | **<0.001** |
| DASS-21 S | 15.90 ± 8.31 | 12.94 ± 8.00 | 14.42 | **<0.001** |
| FQ CC | 2.04 ± 0.61 | 1.91 ± 0.56 | 6.07 | **0.014** |
| FQ EOI | 2.61 ± 0.47 | 2.39 ± 0.52 | 22.01 | **<0.001** |
| FQ TOT | 2.33 ± 0.45 | 2.15 ± 0.47 | 16.69 | **<0.001** |
| AESED AMR | 22.15 ± 9.54 | 18.70 ± 9.53 | 14.30 | **<0.001** |
| AESED RS | 11.08 ± 7.64 | 10.18 ± 6.92 | 1.61 | 0.204 |
| AESED MR | 4.77 ± 5.05 | 5.07 ± 5.14 | 0.37 | 0.543 |
| AESED CF | 9.43 ± 4.20 | 8.69 ± 4.22 | 3.33 | 0.069 |
| AESED TBE | 2.90 ± 3.46 | 2.25 ± 2.96 | 4.33 | **0.038** |
| AESED Overall | 48.82 ± 19.66 | 43.23 ± 20.20 | 8.64 | **0.003** |
| EDSIS N | 16.98 ± 5.46 | 14.73 ± 5.83 | 17.61 | **<0.001** |
| EDSIS DB | 6.62 ± 4.90 | 5.67 ± 4.20 | 4.61 | **0.032** |
| EDSIS G | 9.69 ± 4.61 | 8.17 ± 4.36 | 12.39 | **<0.001** |
| EDSIS SI | 4.90 ± 4.03 | 3.84 ± 3.49 | 8.57 | **0.003** |
| EDSIS Total | 37.50 ± 31.31 | 14.81 ± 14.84 | 19.13 | **<0.001** |

Note: Values are reported as mean ± standard deviation. Group differences between mothers and fathers at baseline (T0) were examined using analyses of variance (ANOVA). F statistics and corresponding p-values are reported. Significant values (p < 0.05) are shown in bold.

*Abbreviations*: DASS-21: Depression, Anxiety and Stress Scale - 21, D: Depression, A: Anxiety, S: Stress; FQ: Family Questionnaire, CC: Critical Comments, EOI: Emotional Overinvolvement, TOT: Total Score; AESED: Accommodation and Enabling Scale for Eating Disorders, AMR: Avoidance and Modifying Routine, RS: Reassurance Seeking, MR: Meal Ritual, CF: Control of Family, TBE: Turning a Blind Eye; EDSIS: Eating Disorders Symptom Impact Scale , N: Nutrition, DB: Dysregulated Behaviour, SI: Social Isolation.

**Table S2. Effects of covariates on caregiver burden in longitudinal mixed-effects models from baseline (T0) to end of workshops (T1).**

|  | Individual with ED Age | | Illness Duration | | Diagnostic Category | | | Caregiver Sex*Time | |
| --- | --- | --- | --- | --- | --- | --- | --- | --- | --- |
|  | Std. β | p-value | Std. β | p-value | Std. β |  | p-value | Std. β | p-value |
| DASS-21 D | 0.03 | 0.587 | -0.06 | 0.100 | -0.12 | 0.361 | | 0.02 | 0.609 |
| DASS-21 A | 0.01 | 0.677 | -0.04 | 0.420 | -0.09 | 0.472 | | 0.04 | 0.222 |
| DASS-21 S | 0.05 | 0.298 | -0.02 | 0.640 | -0.04 | 0.734 | | -0.03 | 0.368 |
| FQ CC | -0.08 | 0.139 | -0.05 | 0.308 | -0.23 | 0.077 | | 0.01 | 0.821 |
| FQ EOI | -0.03 | 0.607 | -0.05 | 0.347 | -0.15 | 0.245 | | -0.03 | 0.612 |
| FQ TOT | -0.06 | 0.227 | -0.06 | 0.259 | -0.23 | 0.081 | | 0.00 | 0.990 |
| AESED AMR | 0.01 | 0.793 | -0.05 | 0.342 | 0.02 | 0.878 | | 0.00 | 0.979 |
| AESED RS | -0.01 | 0.801 | -0.06 | 0.271 | 0.10 | 0.477 | | -0.01 | 0.751 |
| AESED MR | -0.05 | 0.652 | -0.06 | 0.258 | -0.08 | 0.681 | | -0.03 | 0.221 |
| AESED CF | -0.06 | 0.311 | 0.04 | 0.467 | -0.25 | 0.071 | | -0.01 | 0.865 |
| AESED TBE | 0.03 | 0.540 | -0.09 | 0.109 | -0.09 | 0.502 | | 0.01 | 0.666 |
| AESED Overall | 0.00 | 0.964 | -0.06 | 0.288 | -0.01 | 0.916 | | 0.00 | 0.821 |
| EDSIS N | -0.01 | 0.903 | -0.03 | 0.583 | -0.15 | 0.257 | | 0.00 | 0.962 |
| EDSIS DB | 0.06 | 0.291 | 0.00 | 0.968 | -0.12 | 0.393 | | 0.04 | 0.777 |
| EDSIS G | 0.01 | 0.808 | -0.03 | 0.592 | -0.12 | 0.368 | | 0.03 | 0.273 |
| EDSIS SI | 0.01 | 0.893 | -0.03 | 0.601 | -0.02 | 0.750 | | 0.04 | 0.157 |
| EDSIS Total | -0.04 | 0.466 | 0.03 | 0.649 | 0.03 | 0.631 | | 0.03 | 0.190 |

*Note:* Caregiver’s age-adjusted linear mixed-effects models are reported. Standardized beta coefficients and p-values are shown for illness duration, offspring diagnostic category (anorexia nervosa, bulimia nervosa), and the caregiver sex × time interaction, included as fixed effects in models examining longitudinal changes in caregiver outcomes across time.

*Abbreviations*: DASS-21: Depression, Anxiety and Stress Scale - 21, D: Depression, A: Anxiety, S: Stress; FQ: Family Questionnaire, CC: Critical Comments, EOI: Emotional Overinvolvement, TOT: Total Score; AESED: Accommodation and Enabling Scale for Eating Disorders, AMR: Avoidance and Modifying Routine, RS: Reassurance Seeking, MR: Meal Ritual, CF: Control of Family, TBE: Turning a Blind Eye; EDSIS: Eating Disorders Symptom Impact Scale, N: Nutrition, DB: Dysregulated Behaviour, SI: Social Isolation.

**Table S3. Baseline comparisons between caregivers who completed the post-intervention assessment and those lost to follow-up across the main outcome measures.**

|  | Complete  (n=258) | Lost to follow-up  (n=189) | F | p-value |
| --- | --- | --- | --- | --- |
| DASS-21 D | 10.16 ± 7.08 | 11.47 ± 8.55 | 2.60 | 0.107 |
| DASS-21 A | 6.49 ± 6.55 | 7.12 ± 6.87 | 0.87 | 0.352 |
| DASS-21 S | 13.99 ± 7.86 | 14.95 ± 8.51 | 1.32 | 0.251 |
| FQ CC | 1.95 ± 0.55 | 2.00 ± 0.61 | 0.82 | 0.364 |
| FQ EOI | 2.48 ± 0.50 | 2.54 ± 0.50 | 1.14 | 0.287 |
| FQ TOT | 2.22 ± 0.45 | 2.27 ± 0.47 | 1.32 | 0.251 |
| AESED AMR | 19.64 ± 9.49 | 21.19 ± 9.75 | 2.57 | 0.110 |
| AESED RS | 9.81 ± 7.13 | 11.15 ± 7.43 | 3.28 | 0.071 |
| AESED MR | 4.80 ± 4.49 | 4.95 ± 5.36 | 0.09 | 0.761 |
| AESED CF | 9.09 ± 4.00 | 9.13 ± 4.33 | 0.01 | 0.927 |
| AESED TBE | 2.56 ± 3.17 | 2.65 ± 3.31 | 0.07 | 0.785 |
| AESED Overall | 44.41 ± 18.71 | 47.43 ± 20.66 | 2.27 | 0.133 |
| EDSIS N | 15.38 ± 5.67 | 16.33 ± 5.73 | 2.79 | 0.096 |
| EDSIS DB | 6.46 ± 4.68 | 6.09 ± 4.61 | 0.64 | 0.423 |
| EDSIS G | 8.66 ± 4.15 | 9.22 ± 4.74 | 1.53 | 0.216 |
| EDSIS SI | 4.41 ± 3.88 | 4.47 ± 3.83 | 0.02 | 0.879 |
| EDSIS Total | 34.67 ± 14.08 | 34.92 ± 15.64 | 0.03 | 0.868 |

Note: Values are reported as mean ± standard deviation. Group differences between caregivers who completed the post-intervention assessment and those lost to follow-up at baseline (T0) were examined using analyses of variance (ANOVA). F statistics and corresponding p-values are reported.

*Abbreviations*: DASS-21: Depression, Anxiety and Stress Scale - 21, D: Depression, A: Anxiety, S: Stress; FQ: Family Questionnaire, CC: Critical Comments, EOI: Emotional Overinvolvement, TOT: Total Score; AESED: Accommodation and Enabling Scale for Eating Disorders, AMR: Avoidance and Modifying Routine, RS: Reassurance Seeking, MR: Meal Ritual, CF: Control of Family, TBE: Turning a Blind Eye; EDSIS: Eating Disorders Symptom Impact Scale, N: Nutrition, DB: Dysregulated Behaviour, SI: Social Isolation.
