# Supplementary figures and images for: Participation in the New Maudsley Model Workshops is Associated With Reductions in Caregiver Burden in Eating Disorders
Source: Eur Eat Disord Rev. 2026 Apr 10;34(5):1221–30. doi: 10.1002/erv.70111 (PMC13432577; doi:10.1002/erv.70111)

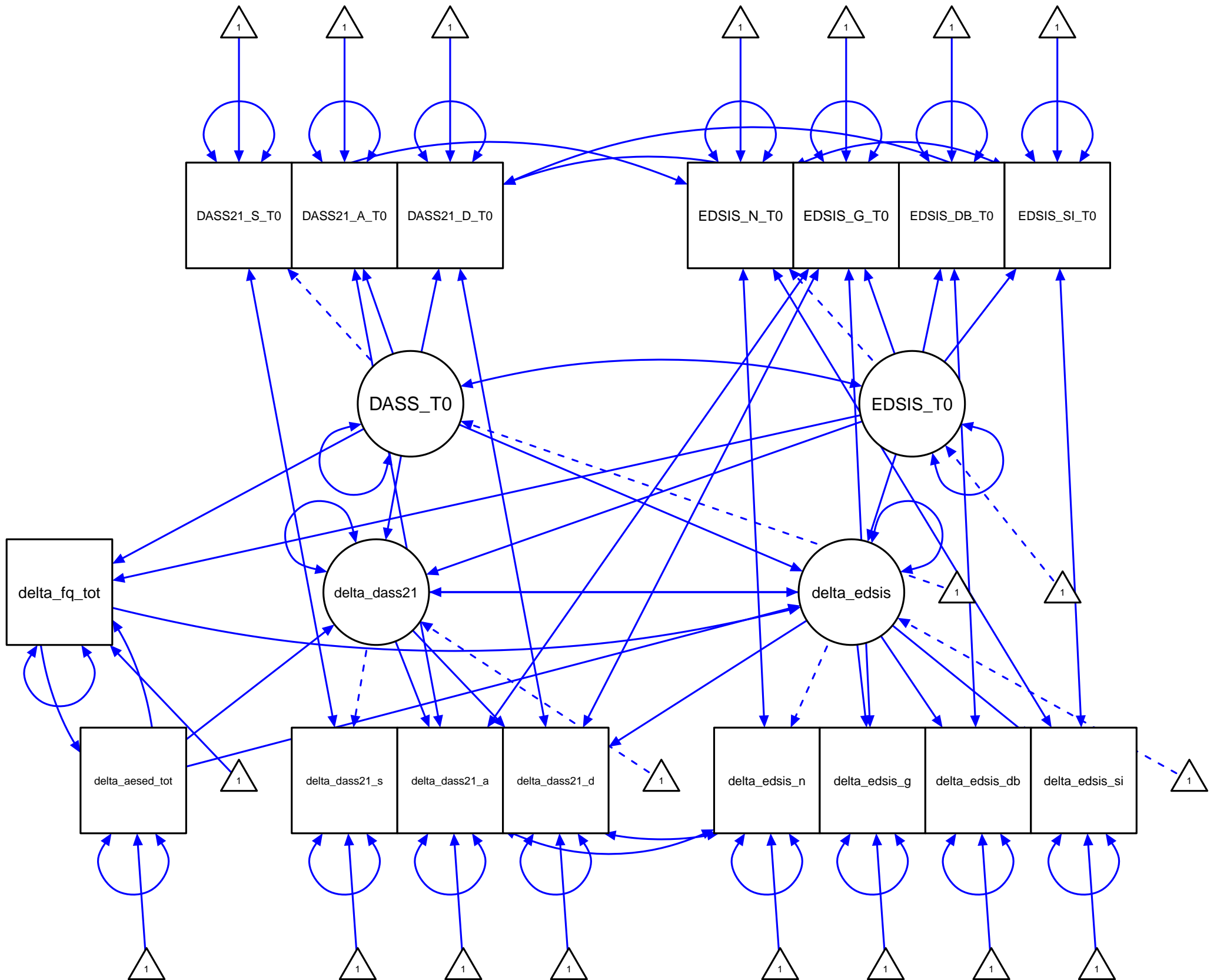

Supplement: Supplementary file 2 — Figure S2: Path diagram illustrating the structural equation model (SEM) examining dynamic associations across caregiver burden domains. [file ERV-34-1221-s001.pdf]
